# Optimal birth weight and term mortality risk differ among different ethnic groups in the U.S.

**DOI:** 10.1038/s41598-019-38583-x

**Published:** 2019-02-07

**Authors:** Jihyun Jeon, Do-Hyun Kim, Min Soo Park, Chang-Gi Park, Sudhir Sriram, Kwang-Sun Lee

**Affiliations:** 1 Department of Pediatrics, CHA Gangnam Medical Center, CHA University School of Medicine, Seoul, Korea; 2 Department of Pediatrics, Dongguk University Ilsan Hospital, Goyang, Korea; 3 Department of Pediatrics, Yonsei University College of Medicine, Seoul, Korea; 4 Department of Pediatrics, University of Chicago Comer Children’s Hospital, Chicago, USA

## Abstract

Among European countries, optimal birth weight at which the mortality is minimal is shown to be different by country. We investigated this difference examining one geopolitical population, the U.S. term live births, born to the five groups of the same ethnic parents; White, Black, Hispanic, North Asian, and South Asian. North Asians and South Asians had more favorable maternal factors for birth weight. Yet, Whites had the highest mean birth weight and South Asians, the lowest. However, neonatal mortality rate in Whites was 0.78 per 1,000 live births, significantly higher than 0.36 and 0.72 per 1,000 live births in North Asians and South Asians, respectively. Other maternal factors hardly explained this ethnic disparity in birth weight or mortality. Optimal birth weight was greatest in Whites (3,890 g), and least in South Asians (3,491 g). However, neonatal mortality at optimal birth weight was significantly lower in North Asians. Adjustment of maternal factors except parental ethnicity changed little of this difference. Optimal birth weight and its mortality differ by ethnicity. On planning the best birth outcome in a population, one should consider the variable mortality risks by ethnicity.

## Introduction

Infant mortality is closely related to both birth weight and gestational age^1,2^. But the birth weight is a stronger predictor for mortality. Susser^3^ reported that when gestational age and birth weight were analyzed simultaneously, birth weight accounted for 90% of the variance of perinatal mortality, whereas gestational age accounted for barely 5%. In general, birth weight is greatest at term gestation, reflecting adequate time for intrauterine growth. However, a large birth weight at term is not necessarily favorable for survival. At term, risk for mortality by birth weight shows a U-shaped curve; the risk is minimal with birth weight at vertex, “optimal birth weight,” and the risk increases either with a decrease or with an increase in birth weight beyond that point.

Many maternal factors influence birth weight, such as ethnicity, age, parity, education, health conditions and her other socioeconomic status. When comparing birth weights of different populations, maternal ethnicity has the strongest influence on birth weight^4,5^. Graafmans and his associates^6^ studied birth outcomes in seven Western European countries, Finland, Sweden, Norway, Denmark, Scotland, the Netherlands, and Belgium. Average birth weight and birth weight at minimal mortality varied among these countries. Compared to the U.S., these European countries may have less heterogeneous ethnic composition, but possibly by country more difference in their socioeconomic status and health care systems. The U.S. population is composed of varied heterogeneous ethnic groups living in one geopolitical area. In our study we asked two questions that (1) whether in one geopolitically defined country, in the U.S., different ethnic groups would have insignificant difference in optimal birth weight, and (2) whether mortality risk would be similar at this optimal birth weight among different ethnic groups. In this study, we wanted to minimize the impact of different provisions of health care system on mortality among different ethnic groups in different countries. Thus, we chose a large birth population of the U.S., where the health care system would have been rather similar across the country.

## Results

### Birth weight(BW), gestational age(GA), and mortality risk

In the U.S., Whites had the highest mean birth weight (3,475 ± 470 g) and South Asians (S. Asians), the lowest (3,228 ± 431 g). However, compared to Whites, South Asians had more favorable maternal factors for birth weight, namely better education and marital status, and lesser smoking and alcohol use, and with less maternal medical complications (Table 1). It is notable that Hispanics had significantly higher mean birth weight than both North Asians (N. Asians) and South Asians, despite their maternal educational level was far lower (>12 years of education, 19% vs. 71% in North Asians and 70% in South Asians), their lower married status (64%, vs, 95% in North Asians and 93% in South Asians), their higher rate of maternal smoking and alcohol use, and their less adequate prenatal care. These observations suggest that term birth weight is most strongly influenced by maternal ethnicity, and little by ethnic differences in many other maternal sociodemographic and health factors (Table 1).

**Table 1:** The Characteristics of the Study Population of Term Singleton Live Births and Their Maternal Characteristics in the U.S., 1995–2006.

	Whites	Blacks	Hispanics	N. Asians	S. Asians
Number	19,018,822	3,086,435	5,905,096	357,926	507,918
BW (g)	3,475 ± 470	3,280 ± 472	3,397 ± 459	3,318 ± 418	3,228 ± 431
GA (wk)	39.1 ± 1.2	39.0 ± 1.2	39.1 ± 1.2	39.2 ± 1.1	39.0 ± 1.1
Male (%)	51.2	50.9	50.8	51.8	51.4
Mortality (per1,000)	0.78	0.98	0.75	0.36	0.72
Maternal age (yr)	27.7 ± 6.7	25.4 ± 7.0	24.9 ± 6.9	30.5 ± 5.7	28.6 ± 6.2
Education >12 yr (%)	62	45	19	71	70
Married (%)	86	48	64	95	93
Smoking (%)	10.7	5.6	4.4	3.7	4.1
Alcohol use (%)	0.8	0.6	0.2	0.1	0.1
Paternal age (yr)	30.9 ± 6.7	28.9 ± 8.4	28.3 ± 7.4	34.3 ± 5.6	33.3 ± 6.2
Prenatal care (%)	67.6	56.3	50.2	59.6	56.2
Maternal RF (%)	25.6	28.1	18.5	17.7	19.5
Neonatal RF (%)	5.5	4.8	3.9	3.7	3.7
Neonatal CA (%)	1.2	1.2	0.8	0.8	0.8

North Asians had the longest mean gestational age (39.2 ± 1.1 wks), while South Asians had the shortest mean gestational age (39.0 ± 1.1 wks). However, this difference in gestational length among all these ethnic groups was less than a day and half at most, hardly accountable for the difference in birth weight among these ethnic groups.

Whites had higher mean birth weight than both North Asians and South Asians. Yet, its neonatal mortality rate was 0.78 per 1,000 live births, significantly higher than 0.36 and 0.72 per 1,000 live births in North Asians and South Asians respectively. Thus, it appears that within term births, maternal ethnicity prevailingly influence the mortality than their birth weights at term.

Then, we further explored the difference in mortality among these five ethnic groups by their maternal sociodemographic and health care factors (Table 2). When not adjusted for these factors, compared to Whites, the risk was least in North Asians (unadjusted odds ratio [OR], 0.47; 95% confidence interval [CI] 0.39–0.55). It also was lesser in Hispanics (unadjusted OR, 0.96, 95% CI 0.92–0.99). However, compared to Whites, this risk was not different in South Asians (unadjusted OR 0.92, 95% CI 0.83–1.02). And it was higher in Blacks (unadjusted OR 1.27, 95% CI 1.22–1.32). Now, adjusted for maternal sociodemographic and health factors, the mortality risks of these five ethnic groups hardly changed (Table 2). This observation indicates that at term birth the maternal ethnicity is the most powerful determinant for the neonatal survival than any other maternal sociodemographic and health factors.

**Table 2:** The Risk of Neonatal Mortality Among Different Ethnic Groups, Compared to Non-Hispanic White Group, Singleton Live Births, the U.S., 1995–2006.

Races	Unadjusted OR	95% CI	Adjusted OR	95% CI
Whites	1.00	—	1.00	—
Blacks	1.27	1.22, 1.32	1.17	1.12, 1.22
Hispanics	0.96	0.92, 0.99	0.84	0.81, 0.87
N. Asians	0.47	0.39, 0.55	0.49	0.41, 0.59
S. Asians	0.92	0.83, 1.02	0.97	0.87, 1.08

For Adjusted OR, the following variables are adjusted; maternal age, parity, education, marital status, medical and obstetric risk factors including alcohol use and smoking, adequacy of prenatal care, and paternal age.

### Optimal birth weight with minimum neonatal mortality

**Figure 1:**
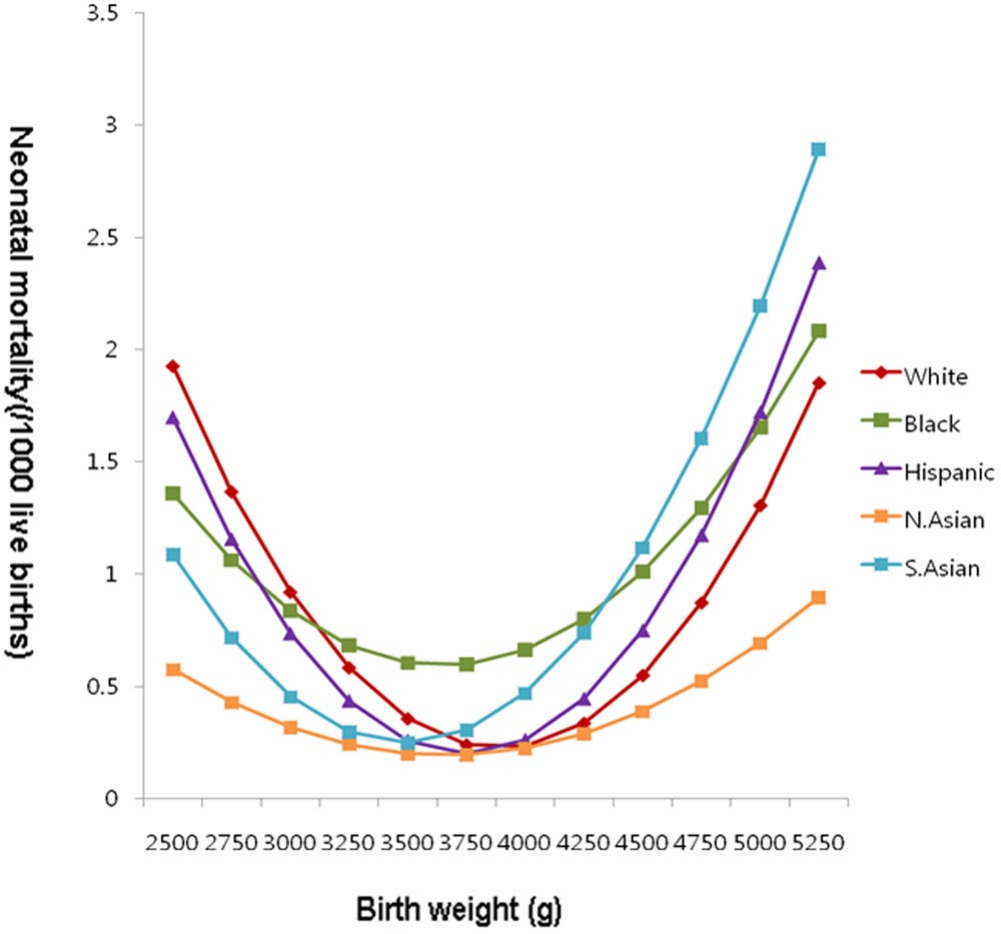
Neonatal mortality risk (/1,000 live births) at term in singleton live births among five ethnic groups in the U.S., 1995–2006. Abbreviations: White: Non-Hispanic White mother and father group; Black: Non-Hispanic black mother and father group; Hispanic: Hispanic mother and father group; N. Asian: Korean mother and father, Japanese mother and father, and Chinese mother and father group; S. Asian: Filipino mother and father, Vietnamese mother and father, and Asian Indian mother and father group.

In all five ethnic groups, the pattern of mortality by birth weight showed a “u-shaped curve”, fitting best to a quadratic equation (Fig. 1). From the derived equation, then we obtained for each racial group the optimal birth weight and mortality risk at this optimal birth weight (Table 3). This analysis brought four notable observations. First, the optimal birth weight differed among the ethnic groups. Second, in all individual ethnic groups the optimal birth weight was greater than its mean birth weight. The third, among all ethnic groups, the lower the mean birth weight, lower the optimal birth weight was. And the fourth, the pattern of mortality risk below and above the optimal birth weight (parabola of the u-shaped curve of mortality risk by birth weight) was not uniform among the ethnic groups (Table 3 and Fig. 1). North Asians had the widest parabola, thus with smaller increment of the mortality risk with decreasing or increasing birth weight from the optimal birth weight, having the most favorable mortality by birth weight distribution, compared to other ethnic groups. The narrow this u-curve, the higher was the overall mortality. But then, one has to know the proportional distribution of birth weights along this u-curve, to attribute differential contributions of two determinants, birth weight distribution and birth weight-specific mortality to the overall crude mortality. Our observation indicates that within term births, different ethnic groups have different birth weight distributions with differing optimal birth weights. Also, mortality risk differs throughout this term birth weight distribution among different ethnic groups.

**Table 3:** The Optimal Birth Weight with Minimum Neonatal Mortality and Their Rates in Five Ethnic Groups, the U.S., 1995–2006.

Races	Whites	Blacks	Hispanics	N. Asians	S. Asians
Optimal birth weight (g)	3,890	3,650	3,745	3,666	3,491
Estimated Minimum mortality (/1000 live births)	0.22	0.59	0.20	0.19	0.25

## Discussion

Mortality in its relationship to birth weight follows a “reversed J” curve^7,8^. This pattern of mortality is universal among all ethnic groups. Crude neonatal or infant mortality rate is determined by its two components, birth weight distribution, particularly a proportion of smaller birth weight groups, and birth weight-specific mortality rates of individual birth weight groups^9,10^. Blacks in the U.S. has higher crude neonatal and infant mortality rate primarily due to its unfavorable birth weight distribution, higher proportion of small birth weight groups, and by lesser, by mortality rate difference in individual birth weight groups^11–14^. However, in the U.S., there is little information on subtle difference in term infants, their birth weight distribution within term gestation and their mortality risk among different ethnic groups.

Platt and his associates^15^ showed that in the U.S., optimal birth weight was higher and mortality risk at term was less in Whites compared to Blacks. When mortality risk was expressed by the relative birth weight (a z-score relative to mean birth weight), they were able to observe consistently high mortality risks in Blacks than in Whites at both below and above the zero z-score. These observations suggest that ethnic disparity in mortality at term could not be solely explained by either mean or optimal birth weight. It is determined by two factors by the ethnicity, (1) differences in proportional birth weight distribution at term, below, at, and above optimal birth weight, and (2) differences in mortality at respective birth weights, below, at, and above optimal birth weight. This concept of mortality at term is explored in our present study.

Graafmans and his associates^6^ studied birth outcomes in seven Western European countries. They reported variable mode of birth weight distribution, ranged between 3,384 g in Flanders and 3,628 g in Finland. The birth weight at the minimal perinatal mortality also differed, but closely related to mean birth weight, about 170 g higher than mean birth weight. Compared to the U.S., Western European countries may have less heterogeneous racial composition, but possibly more differences in their socioeconomic environments and health care systems. In our study, we used the U.S. live birth population. We wanted to study the birth outcomes in more diverse ethnic groups. In the U.S., ethnic identification of births were newly refined and registered starting in the year 1995. To assure that we have an adequate number of live births in all individual ethnic groups, we included our study population of live births for the 12 years, 1995–2006. Thus, we had an adequate number of term singleton live births born to the same ethnic parents, ranging from 19 million births in Whites to half million births in South Asians. In the U.S., over the period, 1995–2006, there had been changes in socioeconomic status and health care system. This study did not examine the potential bias on birth outcomes arising from differential impact of these changes to individual ethnic groups over time. Only using the whole study population for all the years combined, we examined ethnic differences in mean birth weight, gestational age, optimal birth weight, and mortality by term birth weight distribution. As noted by others^16–20^, when controlled for maternal sociodemographic and health care status (age, marital status, parity, education, smoking, alcohol use, and other health care factors including prenatal care status), ethnic differences in birth outcomes changed little. It indicates that the parental ethnicity is the strongest predictor of term birth outcomes, overwhelming all other parental factors.

In essence, our results from the U.S. births with diverse ethnicity confirm the observations made by Graafmans and his associates in seven Western European countries. However, magnitude of difference in mean or modal birth weight in the U.S. ethnic groups was larger, perhaps reflecting its diverse ethnic composition in the U.S. than in the Western European countires. In the present study, Non-Hispanic Whites had the highest mean birth weight followed by Hispanics, the North Asians (Korean, Japanese, and Korean), and non-Hispanic Blacks. The South Asians (Asian Indian, Filipino, and Vietnamese) had the lowest mean birth weight. Within each of individual ethnic groups, maternal sociodemographic and health status is significantly associated with their birth weight outcomes, but little impact was noted when examined the ethnic difference in birth outcomes.

Mortality at the optimal birth weight varied more in the Western European countries than in different ethnic groups in the U.S., possibly reflecting variable health care system in the European countries. Among our studied ethnic groups, the rank order of optimal birth weight precisely followed that of mean birth weight. It was the heaviest in Whites and the lowest in the South Asians. Optimal birth weight in each group was about 8% to 18% higher than its mean term birth weight. These results confirm similar observations made by Graafmans and his associates^6^.

Mortality risk at term by birth weight fits best to a quadratic curve. In this curve, mortality reaches the bottom (µ, the optimal birth weight) and increases more or less symmetrically to either side of the optimal birth weight (σ_p,_ variance of birth weight-specific mortality) (Fig. 1)^21^. The present study showed that mortality risk both at optimal birth weight and at below and above the optimal birth weight differed among the five ethnic groups. Lesser the overall crude mortality rate, lesser the mortality risk was at optimal birth weight and wider was the parabola of the mortality risk curve. Among five ethnic groups, North Asians (Korean, Japanese, and Chinese) had the least overall crude mortality rate with the least mortality risk at optimal birth weight and wider parabola of the mortality curve. This was followed by Hispanics, non-Hispanic Whites, and South and Southeast Asians (Filipino, Vietnamese, and Asian Indian). Non-Hispanic Blacks had the highest overall crude mortality rate with the highest mortality risk at optimal birth weight and the most narrow parabola of the mortality curve.

In conclusion, our present study shows that different ethnic groups in the U.S. have varying mean birth weight at their term gestation. Ethnic difference in mean gestational age at term was little, less than a day and half at most, and was not responsible for the ethnic difference in mean birth weight. Optimal birth weight was about 8% to 18% higher than mean term birth weight in all ethnic groups. Magnitude of mortality risks both at optimal birth weight and at below and above the optimal birth weight was also different among different ethnic groups. Maternal sociodemographic and health factors failed to explain the ethnic difference in all the following measures, mean birth weight, optimal birth weight, and mortality risks at optimal birth weight and at below and above the optimal birth weight. Our study strongly suggests that optimal birth weight and term birth weight distribution of one ethnic group cannot be applied to other ethnic group in planning and delivering the perinatal health care.

## Materials and Methods

### Subjects

Our study population derives from the Birth Cohort Linked Birth/Infant Death Data of the U.S. National Center for Health Statistics (NCHS) from 1995 to 2006. It includes only singleton live births with gestational ages between 37 and 42 weeks (n = 28,876,197). They were born to the five parental groups with both parents with the same ethnicity; (1) non-Hispanic White (Whites) (n = 19,018,822), (2) non-Hispanic Black (Blacks) (n = 3,086,435), (3) Hispanic (Hispanics) (n = 5,905,096), (4) Korean, Japanese, and Chinese (North Asians) (n = 357,926), and (5) Filipino, Vietnamese, and Asian Indian (South Asians) (n = 507,918).

### Definition and data analysis

Neonatal mortality was defined as the number of deaths under 28 days of life per 1,000 live births. Optimal birth weight was defined as the birth weight with the least neonatal mortality at term.

We compared the following variables between the five ethnic groups: mean birth weight, mean gestational age, neonatal mortality, sex, maternal age, education, marital status, smoking, alcohol use, paternal age, adequacy of prenatal care, maternal medical risk factors(RF), neonatal medical risk factors(RF) at birth, neonatal chromosomal and congenital anomalies(CA). In these five ethnic groups, we estimated the optimal birth weight with and without adjustment of the following variables; maternal age, marital status, education, medical risks (anemia, cardiac, acute or chronic lung disease, diabetes, genital herpes, hydramnios/oligohydramnios, hemoglobinopathy, chronic hypertension, preeclampsia, eclampsia, incompetent cervix, previous infant with birth weights more than 4,000 g, previous preterm birth, renal disease, RH sensitization, uterine bleeding, and other medical risk factors), prenatal care, alcohol use, smoking, and paternal age.

### Statistical analysis

Chi-square was used to test for significant difference in maternal characteristics among the ethnic groups. For the assessment of ethnic disparities in the neonatal mortality, we estimated odds ratios with confidence intervals by the multivariable logistic regression. For the estimation of optimal birth weight and birth weight-specific neonatal mortality curve, we used term birth weights by 250 g intervals. The best fit quadratic equation was derived from the data, neonatal mortality (y) and birth weight (x) using the quadratic fit model of CurveExpert 1.3. From this equation, we drew the birth weight-specific mortality curve of the five ethnic groups at term gestation. And also we estimated their individual optimal birth weight with minimal mortality by finding x and y values of vertex. All other statistical analyses, we used Stata (v.13).
